# Fungal-Mediated Biotransformation of the Plant Growth Regulator Forchlorfenuron by *Cunninghamella elegans*

**DOI:** 10.3390/metabo14020101

**Published:** 2024-02-01

**Authors:** Charles M. Moreno, Jaclyn N. Moreno, Matthew C. Valdez, Melinda P. Baldwin, Ana C. Vallor, Paulo B. Carvalho

**Affiliations:** 1 Department of Pharmaceutical Sciences, Feik School of Pharmacy, University of the Incarnate Word, San Antonio, TX 78212, USA; cmoreno5935@outlook.com (C.M.M.); jamoren6@student.uiwtx.edu (J.N.M.); mcvalde1@uiwtx.edu (M.C.V.); mpbaldwi@student.uiwtx.edu (M.P.B.); 2 Department of Biology, School of Mathematics, Science, and Engineering, University of the Incarnate Word, San Antonio, TX 78209, USA; vallor@uiwtx.edu

**Keywords:** forchlorfenuron, cytokinins, *Cunninghamella elegans*, hydroxylation, microbial transformation, liquid chromatography–mass spectrometry, metabolite identification and synthesis

## Abstract

The synthetic cytokinin forchlorfenuron (FCF), while seemingly presenting relatively low toxicity for mammalian organisms, has been the subject of renewed scrutiny in the past few years due to its increasing use in fruit crops and potential for bioaccumulation. Despite many toxicological properties of FCF being known, little research has been conducted on the toxicological effects of its secondary metabolites. Given this critical gap in the existing literature, understanding the formation of relevant FCF secondary metabolites and their association with mammalian metabolism is essential. To investigate the formation of FCF metabolites in sufficient quantities for toxicological studies, a panel of four fungi were screened for their ability to catalyze the biotransformation of FCF. Of the organisms screened, *Cunninghamella elegans* (ATCC 9245), a filamentous fungus, was found to convert FCF to 4-hydroxyphenyl-forchlorfenuron, the major FCF secondary metabolite identified in mammals, after 26 days. Following the optimization of biotransformation conditions using a solid support system, media screening, and inoculation with a solid pre-formed fungal mass of *C. elegans*, this conversion time was significantly reduced to 7 days—representing a 73% reduction in total reaction time as deduced from the biotransformation products and confirmed by LC-MS, NMR spectroscopic data, as well as a comparison with synthetically prepared metabolites. Our study provides the first report of the metabolism of FCF by *C. elegans*. These findings suggest that *C. elegans* can produce FCF secondary metabolites consistent with those produced via mammalian metabolism and could be used as a more efficient, cost-effective, and ethical alternative for producing those metabolites in useful quantities for toxicological studies.

## 1. Introduction

Plant growth regulators (PGRs) are chemicals used to modify the growth and development of plants. PGRs are a very diverse set of compounds divided into several subclasses based on their chemical structure and physiological effects [1,2]. One subclass of PGRs is cytokinins, broadly defined as N6-substituted adenine-based molecules capable of implicating cell division and differentiation [3]. Cytokinins, and phenylurea-derived cytokinins in particular, are a versatile group of agrochemicals that have gained widespread attention in recent years for their anti-cancer, anti-inflammatory, and neuroprotective properties [4,5,6,7]. As a promising class of plant hormones, cytokinins have been found to have an acute dermal toxicity (LD_50_ > 2 g/kg) in Toxicity Category III (slightly toxic) and an oral toxicity (LD_50_ > 5 g/kg) in Toxicity Category IV (practically non-toxic) [8]. However, prolonged exposure to cytokinins has been shown to have adverse effects on the growth and development of selected crops by decreasing the contents of vitamin C and increasing phenolic compound contents [9]. Forchlorfenuron (FCF; 1-(2-chloropyridin-4-yl)-3-phenylurea), a plant growth regulator belonging to the synthetic cytokinin class, was registered in the United States for use on grapes and kiwifruit in 2004 [10,11]. Since then, FCF has been shown to act synergistically with natural auxins to promote fruit size in grapes, kiwifruit, melons, and apples [12,13,14,15]. While the use of these growth enhancements generally leaves residual quantities considered safe for human consumption, previous studies have prompted concern about FCF’s potential mammalian toxicity [16,17,18].

In 2019, a chronic toxicity study of FCF in rats revealed that FCF caused pathological changes in the ovaries, inhibited the proliferation of granulosa cells (GCs) and H295R cells, and downregulated the expression of CYP17A1 and CYP19A1 in estradiol and progesterone production [16]. In addition, a study by Gong et al. reported that FCF induced cardiac morphology deformation, cardiac contractile dysfunction, and erythrocyte reduction in zebrafish [17,19]. In a more recent study evaluating the potential safety risks of FCF on juvenile rats, Ping et al. emphasized the risks of FCF exposure in children after finding that juvenile rats exhibited reduced plasma clearance (CLz) when compared to adult rats [20]. With human populations facing increased exposure to FCF residues through the consumption of fruits and vegetables, these bioaccumulations have also been shown to cause cyto-genotoxicity in human lymphocytes [21,22,23].

**Scheme 1:**
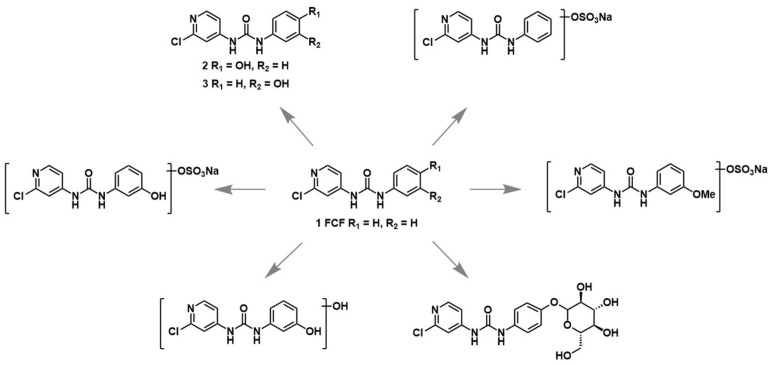
The chemical structure of forchlorfenuron (FCF; 1-(2-chloropyridin-4-yl)-3-phenylurea) and its known metabolites identified in studies with rats as reported by USEPA [10].

Given the potential adverse effects of FCF, understanding its metabolism and the toxicity of its relevant secondary metabolites is essential. To date, six known FCF metabolites have been identified in studies with rats, formed by monosulfonation (forchlorfenuron-sulfate; major metabolite in urine of males and females), monohydroxylation (hydroxy-forchlorfenuron; two monohydroxylated isomers; predominant in feces), monohydroxylation and sulfonation (hydroxy-forchlorfenuron-sulfate), methoxylation and sulfonation (methoxy-forchlorfenuron-sulfate), glucuronidation (forchlorfenuron-glucuronide), and dihydroxylation (dihydroxy-forchlorfenuron) (see Scheme 1) [10].

The metabolism of FCF in rats and mice primarily involves p-hydroxylation of the phenyl moiety, followed by rapid sulfate conjugation and N-glucuronide acid conjugation (in rats) and rapid O-glucuronide acid conjugation (in mice) [24]. While the major metabolic pathways of FCF in mammals involve the hydroxylation of the phenyl ring, additional modifications of the hydroxyl moieties have also been observed to a limited extent [25]. Furthermore, results from in vitro studies with rats and mice indicate that the major metabolite produced is 4-hydroxyphenyl-forchlorfenuron and that there are no qualitative differences in metabolite formation by liver microsomes (rat, mice, and human) [24]. Thus, while the metabolism and toxicokinetics of FCF are well established, our knowledge of the toxicology effects its secondary metabolites have on humans is still limited.

While work has already been published surrounding the identification, synthesis, and safety assessment of FCF secondary metabolites in kiwifruit, no studies have attempted to reproduce FCF’s metabolism in mammals using fungus as a model organism [11]. This major gap in the literature, namely the study of the microbial transformation of FCF for the formation of secondary metabolites and their use to simulate human metabolism, illustrates the need for this work. Microbial transformation is widely accepted as a useful in vitro model to study drug metabolism in mammalian cells [26]. *Cunninghamella elegans*, a filamentous fungus, is an important model organism used in the biotransformation of drugs and other xenobiotics due to its ability to anticipate human metabolism and toxicity [26,27,28,29,30]. Acting as an alternative approach to the mammalian model, the fungal model can reproduce human metabolism and facilitate reactions such as hydroxylation while providing a more efficient, cost-effective, and ethical alternative for conducting metabolic studies [26,30,31,32,33,34,35,36,37,38,39].

Since six metabolites of FCF have been previously identified in mammals, we aimed to confirm the major FCF secondary hydroxylated metabolite found in the literature using the microbial biotransformation of FCF by *C. elegans*. Beyond offering a novel approach to facilitating and optimizing the biotransformation of FCF, this study provides a new shorter synthetic route to obtaining the major FCF secondary hydroxylated metabolites found in the literature. Thus, in the present study, we report on the biotransformation of FCF using a panel of fungi, extraction, isolation, structural elucidation, and a comparison of the biotransformation products with synthesized standards. Our findings provide the first report of the metabolism of FCF by fungi and, therefore, may yield further evidence for the understanding of its metabolism in humans.

## 2. Materials and Methods

### 2.1. Chemicals and Reagents

Forchlorfenuron (1-(2-chloro-4-pyridyl)-3-phenylurea) (purity 98.0%) and high-performance liquid chromatography (HPLC)-grade methanol (≥99.9%), acetonitrile (≥99.9%), and dichloromethane (99.7%) were purchased from Fischer Scientific (Waltham, MA, USA). Hexanes (≥95.0%), ethyl acetate (≥99.5%), acetone (≥99.5%), chloroform (≥99.8%), and isopropyl alcohol (≥99.7%) were purchased from Sigma-Aldrich (Burlington, MA, USA) and were of analytical grade.

Potato dextrose (PD) agar and broth were purchased from Fischer Scientific MP Biochemicals (Solon, OH, USA) and prepared as directed. Yeast mold and yeast malt broth medium were purchased from HiMedia Laboratories (Kennett Square, PA, USA) and prepared as directed. Yeast malt broth and potato dextrose broth were prepared according to the formulation described by Wickerham (Table 1) [31,32]. Bacto Peptone, Yeast Extract, Malt Extract, Potato Extract, and dextrose were purchased from Fischer Scientific (Waltham, MA, USA) and Life Technologies Corporation (Detroit, MI, USA).

### 2.2. Microorganisms and Culture Medium

*Cunninghamella elegans* (ATCC 9245), *Aspergillus brasiliensis* (*niger*) (ATCC 9642), *Rhizopus stolonifer* (ATCC 14037), and *Beauveria bassiana* (ATCC 7159) were purchased from the American Type Culture Collection (Manassas, VA, USA) and provided by the Department of Biology at the University of the Incarnate Word (San Antonio, TX, USA). Fungal strains were maintained on freshly prepared potato dextrose agar plates and subcultured prior to biotransformation.

### 2.3. Culture and Biotransformation Conditions

#### 2.3.1. Standard Conditions

A modified version of the protocol described by Parshikov et al. [33] was used for standard biotransformation conditions. Well-developed fungal mycelia from agar slope cultures were transferred into 250-mL Erlenmeyer flasks (autoclaved at 121 °C for 25 min) containing 100 mL of yeast malt broth medium and incubated for 3 days at 25 °C on a rotary shaker (150 rpm). After 3 days, FCF (50 mg dissolved in 1.0 mL of acetone) was used to dose each of the cultures before resuming incubation under the same conditions for a time trial study at 5, 7, 10, and 14 days for *Aspergillus brasiliensis* (*niger*); 3, 6, and 9 days for *Rhizopus stolonifer*; and 9, 15, 26, and 30 days for *Beauveria bassiana* and *Cunninghamella elegans*. The time trial periods were dependent on fungal strain and based on previously published studies using similar microbial models in other parent compounds [34,35,36,37,38,39,40,41,42,43].

#### 2.3.2. Optimized Conditions

To optimize the yield of metabolites formed during biotransformation, growth conditions for the fungal strain most efficient at catalyzing the biotransformation of FCF were revised, based on the work of Amadio et al. [40], to include a solid support system to increase reaction speed (rate of conversion) and enhance the purification of the isolated metabolite(s). This was followed by a time trial media screening of yeast mold broth versus potato dextrose broth to further accelerate the rate of conversion. These optimized conditions were repeated using an inoculation method with a solid pre-formed fungal mass submerged in potato dextrose broth as a tertiary method to optimize biotransformation.

### 2.4. Extraction and Isolation

Conventional methods commonly used in the extraction and isolation of natural products include solvent extraction, maceration, percolation, and reflux extraction—all of which typically involve the use of large volumes of organic solvents and require a longer extraction time with relatively low extraction yields [44]. Various organic solvents have been shown to extract secondary metabolites from the aqueous layer, with dichloromethane and ethyl acetate being the most commonly used organic solvents for extraction and isolation in biotransformation studies with *Cunninghamella elegans* [27,28,30,42].

However, to enhance the isolation of highly polar FCF secondary metabolites, extraction solvents were selectively altered based on the methods found in Toyohara et al. [45]. Following biotransformation based on a time trial study, the mycelia and filtrates were separated using a Büchner funnel, and the remaining broth was extracted with two equal volumes of dichloromethane (DCM) and a 3:1 chloroform/isopropyl alcohol (IPA) mixture. The solvent was dried over anhydrous sodium sulfate and evaporated in vacuo.

Thin-layer chromatography (TLC) was performed on aluminum plates pre-coated with silica gel G and GF Uniplates (Analtech) in the solvent system EtOAc/hexanes (50:50). All metabolites were purified by semi-preparative flash chromatography carried out on silica gel 60 (Scientific Adsorbents Incorporated; particle size 32–63 µm, pore size 60 Å) using 0–100% EtOAc/hexanes solvent system in gradient mode, eluting from 0 to 30% DCM/3M NH_3_ in methanol. The residue was then dissolved in 2 mL of acetonitrile for HPLC analysis on a Dionex Ultimate 3000 HPLC system.

### 2.5. Identification of Metabolites

Metabolites were tentatively identified by LC-MS on an Agilent 6320 ESI-TOF LC-MS system and an ISQ EM Single Quadrupole mass spectrometer. ^1^H and ^13^C NMR spectra were recorded on a Bruker DPX 300 operating at 400 MHz and 100 MHz at ambient temperature. The samples were dissolved and prepared in deuterated solvent (DMSO-*d_6_*), with residual solvents being used as an internal standard. Spectra were acquired in positive ion mode and analyzed using Bruker TopSpin Software (Version 4.2).

### 2.6. Synthesis of Metabolites

#### 2.6.1. General Procedure for Preparation of Reference Standards

The synthesis of our proposed metabolites has been previously published by the Yue group [2,11]. However, we envisioned a new shorter synthetic route that did not make use of protecting groups and eliminated column chromatography. Our synthetic route began with commercially available 2-chloroisonicotinic acid. This carboxylic acid was treated with DPPA to produce the common intermediate isocyanate, which was subsequently reacted with 3&4-aminophenol to provide our proposed metabolites, 4-hydroxyphenyl-forchlorfenuron and 3-hydroxyphenyl-forchlorfenuron, in an acceptable yield.

#### 2.6.2. Characterization Data

*1-(2-chloropyridin-4-yl)-3-(4-hydroxyphenyl)urea*

**4-hydroxyphenyl-forchlorfenuron**


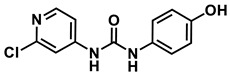


^1^H NMR (400 MHz, DMSO) δ 9.25 (s, 1H), 9.16 (s, 1H), 8.65 (s, 1H), 8.15 (d, *J* = 5.7 Hz, 1H), 7.63 (d, *J* = 1.9 Hz, 1H), 7.29 (dd, *J* = 5.7, 1.9 Hz, 1H), 7.26–7.17 (m, 2H), 6.74–6.62 (m, 2H). ^13^C NMR (100 MHz, DMSO) δ 153.68, 152.48, 151.35, 150.33, 149.85, 130.52, 121.55, 115.69, 112.19, 111.49.

*1-(2-chloropyridin-4-yl)-3-(3-hydroxyphenyl)urea*

**3-hydroxyphenyl-forchlorfenuron**


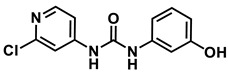


^1^H NMR (400 MHz, DMSO) δ 9.37 (s, 1H), 9.25 (s, 1H), 8.85 (s, 1H), 8.17 (d, *J* = 5.5 Hz, 1H), 7.63 (d, *J* = 2.0 Hz, 1H), 7.30 (dd, *J* = 5.6, 2.0 Hz, 1H), 7.06 (t, *J* = 8.1 Hz, 1H), 7.02 (t, *J* = 2.3 Hz, 1H), 6.84–6.77 (m, 1H), 6.46–6.37 (m, 1H). ^13^C NMR (100 MHz, DMSO) δ 158.23, 152.15, 151.39, 150.42, 149.61, 140.26, 129.99, 112.31, 111.64, 110.29, 109.84, 106.20.

## 3. Results and Discussion

Given that filamentous fungi are a major source of functional secondary metabolites, the four fungal strains—*Aspergillus brasiliensis* (*niger*), *Rhizopus stolonifer*, *Beauveria bassiana*, and *Cunninghamella elegans*—were explored as promising model organisms to catalyze the biotransformation of FCF because of their unique ability to produce secondary metabolites in other parent compounds [35,36,38,39,40]. These recent advances in using microorganisms to study metabolic processes in humans is particularly significant because fungi have enzymatic systems similar to those of mammalian cells [26,46]. As an inexpensive and readily available alternative to other metabolic studies, this method has widespread clinical applications for quickly evaluating the formation of potentially toxic forchlorfenuron metabolites.

Despite the major advantages of microbial transformation procedures, it is widely known that not all secondary metabolites are produced using standard cultivation conditions, so this study evaluated optimized conditions for growth media composition, including the use of a solid support system, media screening, and inoculation with a solid pre-formed fungal mass. These optimized conditions were subsequently used with the fungal strain most efficient at catalyzing the biotransformation of FCF to uncover the potential of fungi to produce FCF secondary metabolites consistent with those produced in mammalian metabolism [43].

### 3.1. Rapid Screening Results from Fungal Panel under Standard Conditions

Following standard cultivation conditions, isolated forchlorfenuron secondary metabolites were analyzed based on a quick screen of metabolization according to high-performance liquid chromatography (HPLC) retention times after a time trial study. Time trial data for each fungal strain were measured at 5, 7, 10, and 14 days for *Aspergillus brasiliensis* (*niger*); 3, 6, and 9 days for *Rhizopus stolonifer*; and 9, 15, 26, and 30 days for *Beauveria bassiana* and *Cunninghamella elegans* based on previously published studies using similar microbial models in other parent compounds [34,35,36,37,38,39,40,43].

**Figure 1:**
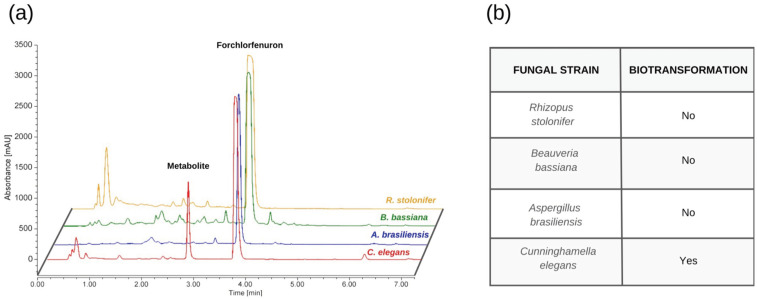
(**a**) HPLC chromatogram overlay of biotransformation results after 26 days showing metabolite formation from *C. elegans* under standard conditions. (**b**) Interpretation of biotransformation results for each fungal strain.

As shown in the HPLC chromatogram overlay presented in Figure 1, *Aspergillus brasiliensis* (*niger*), *Rhizopus stolonifer*, and *Beauveria bassiana* showed negligible metabolic activity, while *Cunninghamella elegans* significantly metabolized FCF to one metabolite at Day 26, with no significant increase in metabolite production being detected at Day 30.

**Figure 2:**
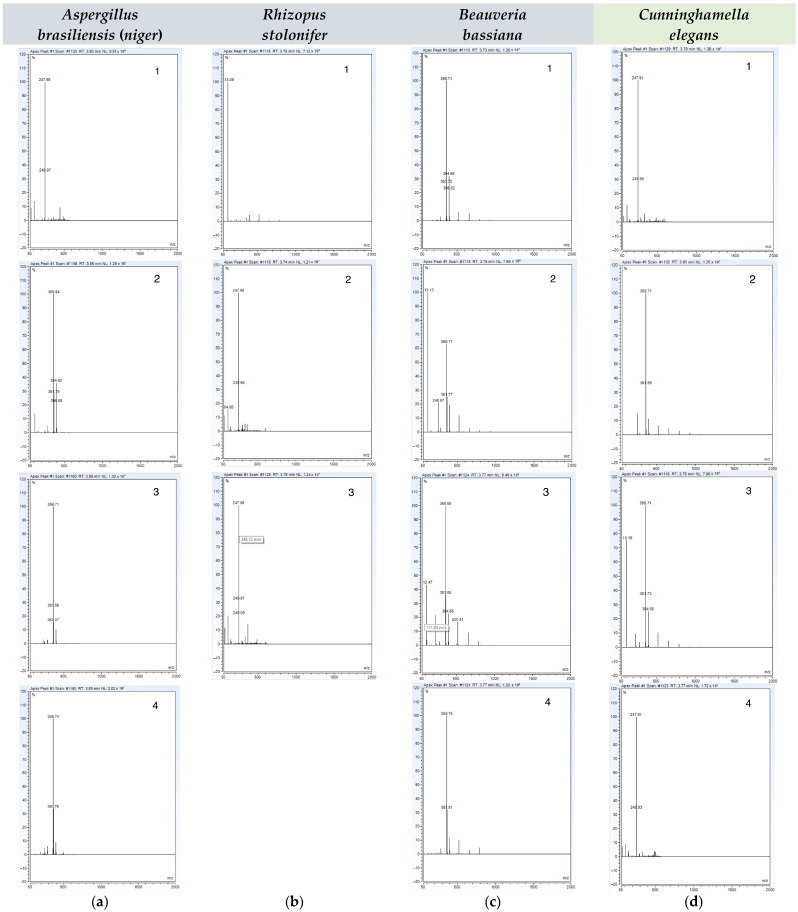
High-resolution tandem mass spectrometry (HR-MS) spectra reporting the biotransformation of FCF by (**a**) *Aspergillus brasiliensis* (*niger*) at 5 days (**1**), 7 days (**2**), 10 days (**3**), and 14 days (**4**); (**b**) *Rhizopus stolonifer* at 3 days (**1**), 6 days (**2**), and 9 days (**3**); (**c**) *Beauveria bassiana* at 9 days (**1**), 15 days (**2**), 26 days (**3**), and 30 days (**4**); and (**d**) *Cunninghamella elegans* at 9 days (**1**), 15 days (**2**), 26 days (**3**), and 30 days (**4**). Fungal strains not capable of catalyzing the biotransformation of FCF are presented in blue, while the fungal strain capable of catalyzing the biotransformation of FCF is presented in green.

The initial detection of this metabolite was confirmed based on our HPLC analysis of the extract from the culture broth, in which the residual or non-transformed forchlorfenuron eluted at 3.9 min, and one metabolite eluted at 2.6 min. From these results, it was determined that the isolated metabolite had a shorter retention time than that of the parent compound, indicating that the fungus produced a metabolite with higher polarity. Time trial HPLC data and high-resolution tandem mass spectrometry spectra for each fungal strain can be found in Figure S3 and Figure 2.

Among the four tested fungal strains, *Cunninghamella elegans* was the only fungus capable of catalyzing the biotransformation of FCF. However, under standard conditions, the time to metabolize FCF was 26 days, which was not sufficient at mimicking the metabolism of FCF in mammals in a timeframe to offer substantial benefit. As a result, *C. elegans* was retained and used for further testing under optimized conditions to increase the rate of conversion from biotransformation.

### 3.2. Optimization of Biotransformation Conditions

#### 3.2.1. Solid Support System

To optimize the 26-day biotransformation of FCF by *C. elegans*, a solid support system was used to potentially increase reaction speed (rate of conversion) and enhance the purification of the isolated metabolite. As cited in previously published studies, immobilized growth is an efficient method to increase biotransformation product yields and facilitate biofilm growth [40]. The solid support system used in this study was accomplished through the addition of porous substrates (glass wool or cotton).

**Figure 3:**
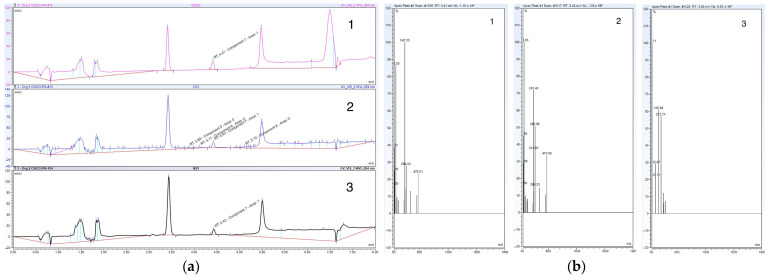
(**a**) High-performance liquid chromatography (HPLC) chromatograms reporting little to no optimization using solid support systems glass wool **1** and cotton **2** compared to normal **3** at 21 days; (**b**) high-resolution tandem mass spectrometry (HR-MS) spectra reporting little to no optimization using solid support systems glass wool **1** and cotton **2** compared to normal **3** at 21 days.

Based on the HPLC chromatograms and HR-MS spectra presented in Figure 3, the use of immobilized cultures as a solid support system minimally increased the rate of conversion from 26 days to 21 days. However, despite a minimal increase in the rate of conversion, it was noted that the addition of glass wool enhanced the ease of metabolite extraction and purification. Additionally, the use of these porous materials as substrates for biofilm growth decreased contamination incidences (i.e., contamination incidences decreased when using glass wool or cotton).

#### 3.2.2. Media Screen

**Figure 4:**
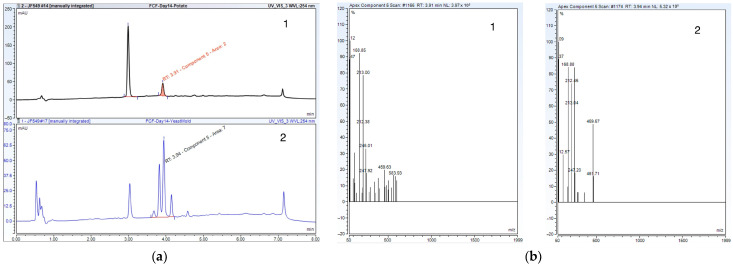
(**a**) High-performance liquid chromatography (HPLC) chromatograms reporting an accelerated rate of conversion from parent to metabolite when comparing the media screening results of potato dextrose broth **1** and yeast mold broth **2** at 14 days; (**b**) high-resolution tandem mass spectrometry (HR-MS) spectra reporting an accelerated rate of conversion from parent to metabolite when comparing the media screening of potato dextrose broth **1** and yeast mold broth **2** at 14 days.

A lack of substantial optimization from the solid support system prompted us to perform a media screen comparing outcomes from yeast malt broth to yeast mold broth, and potato dextrose broth as a secondary method, to accelerate the rate of conversion. A time trial study of both yeast mold broth and potato dextrose broth showed an accelerated rate of conversion from 26 days (standard conditions) and 21 days (solid support system) to 14 days post-media screen. By selecting yeast mold broth or potato dextrose broth as the optimal liquid media for the biotransformation of FCF by *C. elegans*, we were able to reduce the overall transformation time by 7 days. The potato dextrose broth produced a cleaner result with smaller trace amounts of residual or non-transformed drug compared to yeast mold broth (see Figure 4); therefore, it was used in a subsequent inoculation study involving the use of a solid pre-formed fungal mass of *C. elegans*.

#### 3.2.3. Inoculation with Solid Pre-Formed Fungal Mass of *C. elegans*

**Figure 5:**
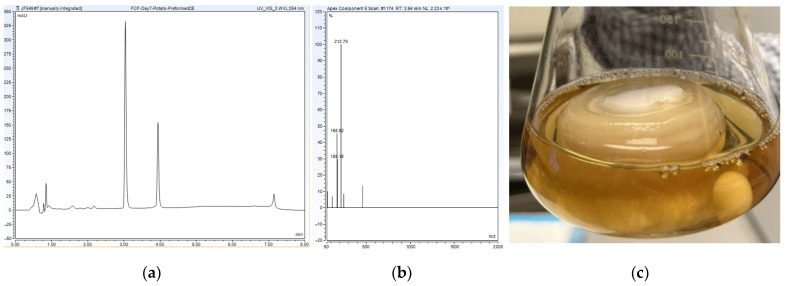
(**a**) High-performance liquid chromatography (HPLC) chromatogram reporting an accelerated rate of conversion from parent to metabolite using a pre-formed fungal mass of *C. elegans* in potato dextrose broth at 7 days; (**b**) high-resolution tandem mass spectrometry (HR-MS) spectra reporting an accelerated rate of conversion from parent to metabolite using a pre-formed fungal mass of *C. elegans* in potato dextrose broth at 7 days; (**c**) pre-formed fungal mass of *C. elegans* submerged in potato dextrose broth.

As a tertiary method to optimize the biotransformation of forchlorfenuron by *C. elegans*, an inoculation method with a solid pre-formed fungal mass of *C. elegans* submerged in potato dextrose broth was used to further increase the rate of conversion from parent drug to metabolite. As depicted in Figure 5, the inoculation method dramatically increased the rate of conversion from 14 days to 7 days by using a pre-formed fungal mass of *C. elegans* submerged in potato dextrose broth—representing a 50% reduction in reaction time to fully metabolize the parent drug. In all, this inoculation method, involving a solid pre-formed fungal mass of *C. elegans* submerged in the optimal potato dextrose broth, provides a highly efficient approach to metabolize FCF via *C. elegans* over a time period that mimics metabolism in the mammalian system.

#### 3.2.4. Optimization Analysis and Percentage Change

The optimization of biotransformation conditions through the use of a solid support system, media screening, and inoculation with a solid pre-formed fungal mass of *C. elegans* significantly increased the rate of conversion from parent drug to metabolite, as calculated from the percent change in biotransformation time. This percent change in conversion time was calculated after each stepwise optimization process to determine the extent of optimization.

As reported herein, the standard biotransformation conditions resulted in a 26-day conversion time, which was reduced to 21 days upon the addition of a solid support system. This optimization process resulted in a 19% decrease in reaction time to convert FCF to its metabolite form. While this process offered slight optimization, 21 days was still not sufficient to offer a substantial advantage when compared to traditional metabolic studies. Upon further optimization using media screening, the 21-day conversion time from the use of a solid support system was further decreased to 14 days upon selecting the best growth media. The selection of potato dextrose broth as the optimal growth media resulted in a 33% decrease in reaction time to convert FCF to its metabolite form. This 14-day conversion time from optimal media selection was decreased significantly to 7 days when inoculated with a solid pre-formed fungal mass of *C. elegans*, resulting in a 50% decrease in reaction time to convert FCF to its metabolite form.

**Figure 6:**
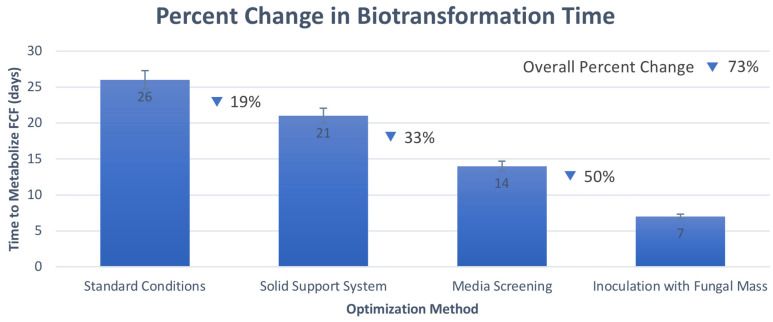
Percent change in biotransformation time with each stepwise optimization process.

Compared to the 26-day conversion time under standard conditions, the use of a solid support system, potato dextrose broth, and inoculation with solid pre-formed fungal mass of *C. elegans* generated optimized conditions for growth with a final conversion time of 7 days, which resulted in an overall percent change of 73%. This significant reduction in conversion time provides a much more efficient, cost-effective, and ethical alternative to catalyze the biotransformation of FCF. Calculations for the percent change from each stepwise optimization process are reported in Table S1, and these percentage decreases in conversion time are visualized in Figure 6. These optimized growth techniques have not been applied to the biotransformation of FCF in previously published studies, thus adding to the novelty of this study.

### 3.3. Identification and Structural Elucidation of the Isolated Metabolite

The forchlorfenuron metabolite profile identified in mammals encompasses hydroxylated, dehydroxylated, and glucuronidated forms of FCF. As reported, the major FCF secondary hydroxylated metabolites 4-hydroxyphenyl-forchlorfenuron and 3-hydroxyphenyl-forchlorfenuron have been previously reported in kiwifruit and oriental melon [11,44,47,48]. However, these metabolites and their associations with human metabolism have not been extensively studied. Given recent concerns about the toxicity of these metabolites, specifically regarding significant cytotoxicity against Chinese hamster ovary cells, this study meets the need to study the formation of these metabolites using model organisms that mimic human metabolism [11,45].

**Figure 7:**
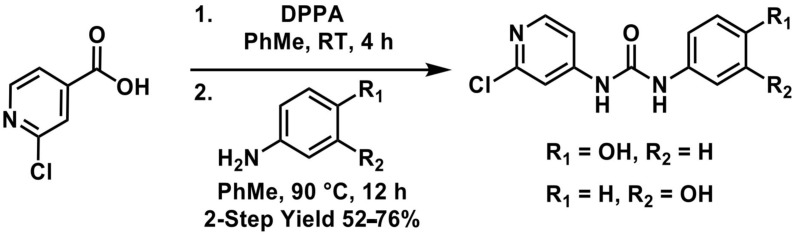
Synthetic route to isolated FCF secondary hydroxylated metabolites 4-hydroxyphenyl-forchlorfenuron and 3-hydroxyphenyl-forchlorfenuron.

Based on the identification of the FCF secondary hydroxylated metabolites found in the literature, isolated metabolites from the biotransformation study were tentatively identified by comparing the HPLC chromatograms, HR-MS spectra, and retention times of potential metabolites with the parent compound forchlorfenuron and a synthesized reference standard of the FCF secondary hydroxylated metabolites, 4-hydroxyphenyl-forchlorfenuron, and 3-hydroxyphenyl-forchlorfenuron. The structure of the isolated metabolites was proposed in accordance with their fragmentation in HR-MS/MS, and the synthetic route to the isolated FCF secondary hydroxylated metabolites is depicted in Figure 7.

This synthetic route offers a new and shorter pathway to obtaining these metabolites that has not been previously published. In the literature, the synthesis of these FCF secondary hydroxylated metabolites using the BBr_3_ demethylation reaction has been proposed [11]. This reaction has been previously carried out in two steps, over 32 h, and with the use of column chromatography [11]. The synthetic route used in this study can be carried out in two steps, over 16 h, and without the use of column chromatography. This unique approach to generating the synthesized metabolites in a shorter timeframe and without the use of column chromatography is especially useful for future studies evaluating the synthesis of FCF secondary hydroxylated metabolites.

**Figure 8:**
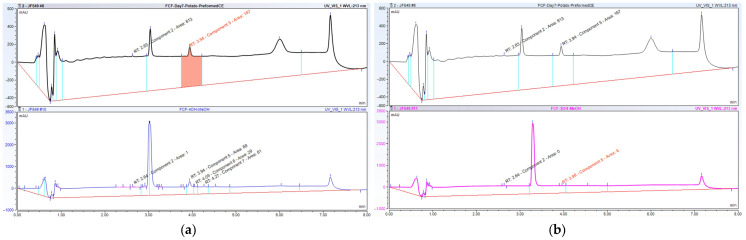
(**a**) High-performance liquid chromatography (HPLC) chromatogram overlay reporting the confirmation of the major FCF secondary metabolite (4-hydroxyphenyl-forchlorfenuron) with an isolated metabolite found in the optimized biotransformation study involving the use of inoculation with a solid fungal mass of *C. elegans* at 7 days; (**b**) high-performance liquid chromatography (HPLC) chromatogram overlay reporting a lack of the minor FCF secondary metabolite (3-hydroxyphenyl-forchlorfenuron) in the optimized biotransformation study involving the use of inoculation with a solid fungal mass of *C. elegans* at 7 days.

The synthesized standards of the FCF secondary hydroxylated metabolites (4-hydroxyphenyl-forchlorfenuron and 3-hydroxyphenyl-forchlorfenuron) were fully characterized (^1^H and ^13^C NMR, HR-MS), and their HR-MS/MS spectral data were compared to those obtained in the biotransformation study. HPLC analyses of the biotransformation product(s) revealed that the isolated metabolite had the same ultraviolet (UV) spectral data and retention time as the major FCF secondary hydroxylated metabolite, 4-hydroxyphenyl-forchlorfenuron, identified in mammals, as noted in Figure 8 and Table 2. Though the minor FCF secondary hydroxylated metabolite, 3-hydroxyphenyl-forchlorfenuron, was synthesized for analysis, this metabolite was not detected among the isolated metabolite(s) in the biotransformation study with *C. elegans*.

**Figure 9:**
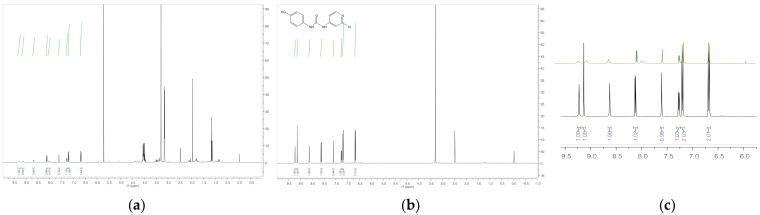
(**a**) ^1^H NMR spectra of Fraction 66 isolated metabolite; (**b**) ^1^H NMR spectra of synthetic hydroxylated metabolite; (**c**) ^1^H NMR spectra overlay of (**a**,**b**) isolated metabolite (in green) and synthetic hydroxylated metabolite (in black) showing an enhanced view of the aromatic region and confirmation of the isolated metabolite structure.

High-resolution mass spectrometry of the isolated metabolite exhibited ions at *m*/*z* 263.7 [M + H] +, respectively, which is consistent with the introduction of a hydroxyl group. As the major FCF secondary hydroxylated metabolite of forchlorfenuron, 4-hydroxyphenyl-forchlorfenuron, was tentatively identified as the major isolated metabolite after our evaluation of the ^1^H and ^13^C-NMR spectra, which confirmed the hydroxyl moiety at the 4-position and established its molecular formula as C_12_H_10_ClN_3_O_2_. These findings were also supported by nearly identical chemical shift and coupling constant values compared to the synthetic reference standard, as depicted in Figure 9. When evaluated together, these data definitely identified the metabolite isolated in our biotransformation study as 4-hydroxyphenyl-forchlorfenuron.

## 4. Conclusions

Together, these results confirmed the structure of the isolated metabolite as the major FCF secondary metabolite in mammals, 4-hydroxyphenyl-forchlorfenuron, formed by optimized biotransformation of the parent drug by *C. elegans* at a detection time of 7 days. It is worth noting that the structural elucidation of our isolated metabolite, 4-hydroxyphenyl-forchlorfenuron, is consistent with studies previously found in the literature and related studies performed by Zhang et al., Shan et al., and Wang et al., wherein they also made tentative identifications of derived forchlorfenuron hydroxylated metabolites from kiwifruit and oriental melon [2,10,11,44,47,48]. However, despite the recent advances made in elucidating the metabolic fates of forchlorfenuron, this study fills three important gaps in the literature.

First, contrary to the methods for FCF metabolite synthesis published in prior studies, this study provides a new shorter synthetic route to synthesizing the two hydroxylated FCF secondary metabolites found in the literature. As discussed herein, this new method decreases the time to obtain the hydroxylated FCF secondary metabolites in the literature from 32 h to 16 h [11]. Additionally, the synthetic route depicted in this study does not make use of protecting groups and eliminates column chromatography, both of which expand upon the methodology reported in the literature in the search for hydroxylated FCF secondary metabolites.

Second, this study showcases the diverse application of fungus as a model organism to test FCF metabolism pathways while furthering our understanding of mammalian metabolism. As a result, these findings provide the first report of the biotransformation of the plant growth regulator forchlorfenuron by the fungus *Cunninghamella elegans*, which offers a promising method to accurately and efficiently reproduce the metabolism of FCF in mammals. The most relevant findings from the biotransformation of FCF by *C. elegans* were that the parent compound produced a structural isomer of hydroxylated-forchlorfenuron at the 4-position with retention time, mass spectra, and NMR spectra matching those of the major FCF secondary metabolite previously identified in mammals (4-hydroxyphenyl-forchlorfenuron).

Finally, this study also features biotransformation optimization techniques which have not been previously applied to FCF. These techniques included the use of a solid support system, media screening, and inoculation with a solid pre-formed fungal mass of *C. elegans*. As a result of these methods, over the course of this study, the biotransformation process was significantly improved from a 26-day conversion time (standard conditions) to a 7-day conversion time (optimized conditions)—representing a 73% reduction in the total reaction time.

Thus, beyond providing the first report of the biotransformation of forchlorfenuron by *C. elegans* and confirmation of the major FCF secondary metabolite (4-hydroxyphenyl-forchlorfenuron) found in mammals, this study provides an attractive alternative to traditional metabolic studies or organic synthesis, as biotransformation can be used as a more efficient, cost-effective, and ethical alternative for predicting the metabolism of FCF in mammals.

In conjunction with its potential for use in future studies, *C. elegans* can fully metabolize FCF over a 7-day period, meaning that incubations can be scaled up, purified, and further characterized on a larger scale to elucidate the metabolic fates of other organic compounds. As an experimental basis for future research centering around forchlorfenuron, its metabolites, and other plant growth regulators in the agricultural industry, our study provides valuable information for predicting FCF secondary metabolites which may be useful for further research into the toxicity and potential adverse effects of these secondary metabolites in humans.

## Figures and Tables

**Table 1:** Media formulation for biotransformation study.

Ingredient	Yeast Malt Broth (in L or g)	Potato Dextrose Broth (in L or g)
Distilled Water	1 L	1 L
Bacto Peptone	5 g	---
Yeast Extract	3 g	---
Malt Extract	3 g	---
Potato Extract	---	4 g
Dextrose	10 g	20 g

**Table 2:** Forchlorfenuron and its isolated metabolite retention time (RT), detected through the biotransformation of FCF by *C. elegans*. Data were measured using the UltiMate 3000 system (Thermo) and an ISQ EM Single Quadrupole Mass Spectrometer (Thermo).

Compound	Formula	Retention Time (min)	[M + H] + *m*/*z*	Type of Transformation
FCF ^1^ 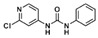	C_12_H_10_ClN_3_O	3.94	247.7	—
4-OH-FCF ^2^ 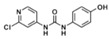	C_12_H_10_ClN_3_O_2_	2.63	263.7	hydroxylation
3-OH-FCF ^3,^* 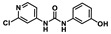	C_12_H_10_ClN_3_O_2_	3.38	263.7	hydroxylation

^1^ Forchlorfenuron; 1-(2-chloropyridin-4-yl)-3-phenylurea; ^2^ 4-hydroxyphenyl-forchlorfenuron, 1-(2-chloropyridin-4-yl)-3-(4-hydroxyphenyl)urea; ^3^ 3-hydroxyphenyl-forchlorfenuron, 1-(2-chloropyridin-4-yl)-3-(3-hydroxyphenyl)urea. * Metabolite synthesized but not isolated in the biotransformation study.

## Data Availability

The data presented in this study are available in the article and Supplementary Materials.

## Supplementary Materials

The following supporting information can be downloaded at: https://www.mdpi.com/article/10.3390/metabo14020101/s1, Figure S1: HPLC chromatogram and HR-MS spectra for pure forchlorfenuron; Figure S2: HPLC chromatogram and HR-MS spectra for *C. elegans* control; Figure S3: High-performance liquid chromatography (HPLC) chromatograms reporting the biotransformation of FCF by fungal strains; Table S1: Percent change in biotransformation time following optimization; Figure S4: HPLC chromatogram of pure forchlorfenuron, 4-hydroxyphenyl-forchlorfenuron, and 3-hydroxyphenyl-forchlorfenuron overlay; Figure S5: ^1^H and ^13^C NMR data for the isolated metabolite (4-hydroxyphenyl-forchlorfenuron) found in the optimized biotransformation study; Figure S6: ^1^H and ^13^C NMR data for the synthetic metabolites used as reference standards (4-hydroxyphenyl-forchlorfenuron and 3-hydroxyphenyl-forchlorfenuron).
