# Supplementary material for: Fungal-Mediated Biotransformation of the Plant Growth Regulator Forchlorfenuron by Cunninghamella elegans
Source: Metabolites. 2024 Feb 1;14(2):101. doi: 10.3390/metabo14020101 (PMC10890479; doi:10.3390/metabo14020101)
Supplement: Supplementary file 1 [file metabolites-14-00101-s001.zip › metabolites-2757284-supplementary.pdf]

metabolites

Supplementary Materials

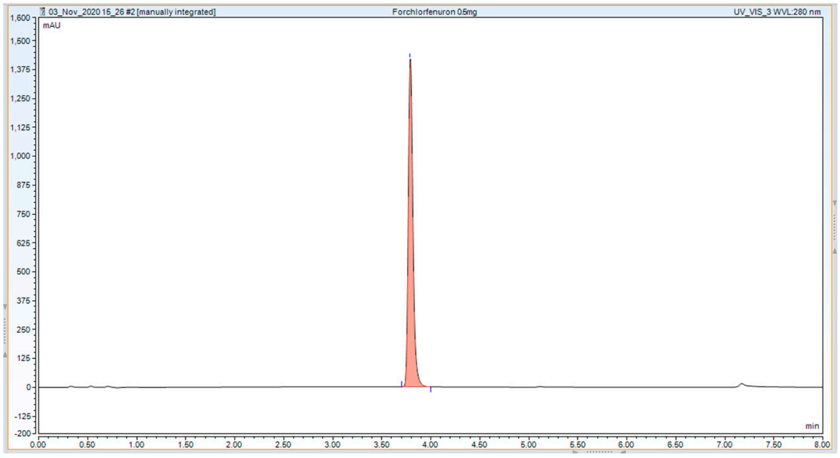

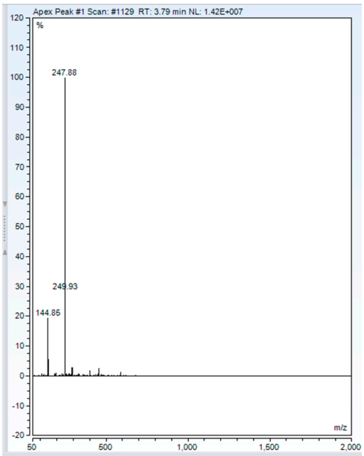

(a) (b)

Figure S1. HPLC chromatogram and HR-MS spectra for pure forchlorfenuron.

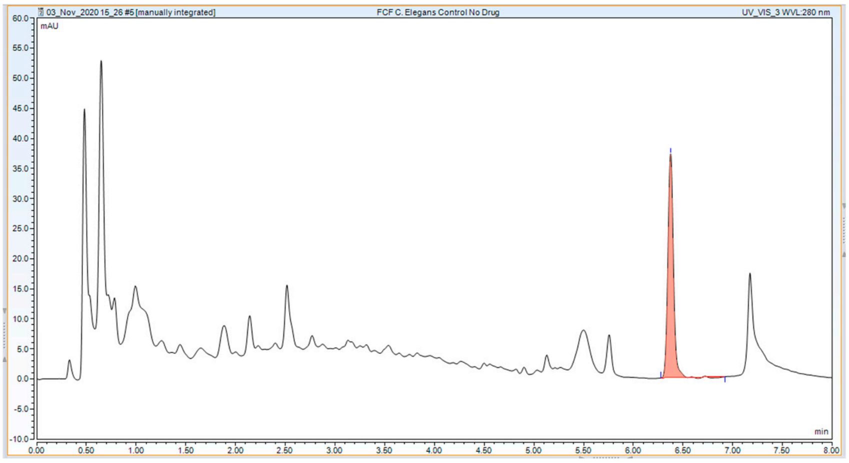

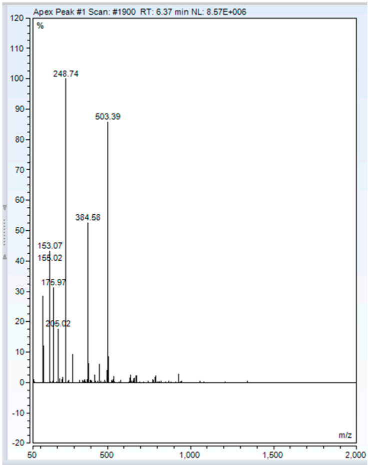

(a) (b)

Figure S2. HPLC chromatogram and HR-MS spectra for *C. elegans* control.

Aspergillus brasiliensis (niger)	*Rhizopus stolonifer*	*Beauveria bassiana*	*Cunninghamella elegans*

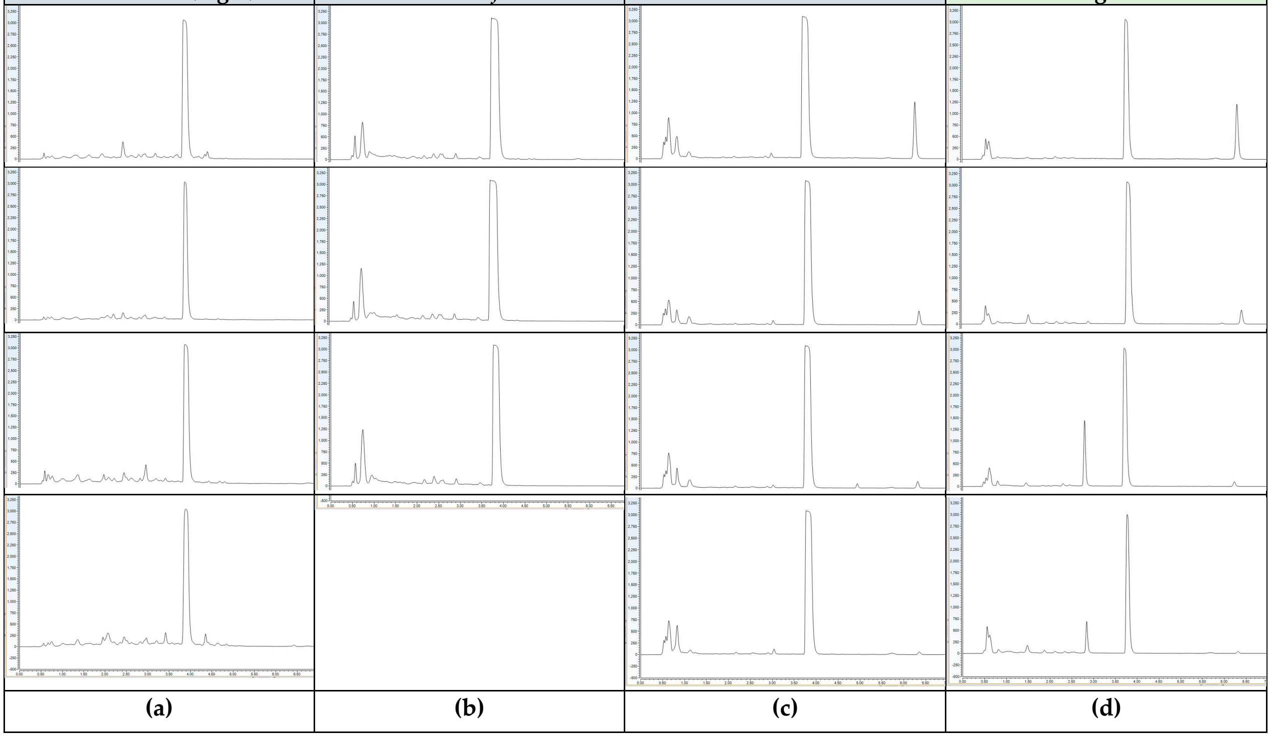

(a)	(b)	(c)	(d)

Figure S3. High-performance liquid chromatography (HPLC) chromatograms reporting biotransformation of FCF by fungal strains. High-performance liquid chromatography (HPLC) chromatograms reporting biotransformation of FCF by **(a)** *Aspergillus brasiliensis (niger)* at 5 days (**1**), 7 days (**2**), 10 days (**3**), and 14 days (**4**); **(b)** *Rhizopus stolonifer* at 3 days (**1**), 6 days (**2**), and 9 days (**3**); **(c)** *Beauveria bassiana* at 9 days (**1**), 15 days (**2**), 26 days (**3**), and 30 days (**4**); (d) *Cunninghamella elegans* at 9 days (**1**), 15 days (**2**), 26 days (**3**), and 30 days (**4**).

Table S1. Percent Change in Biotransformation Time Following Optimization.

The percent change in biotransformation time was calculated according to the following percent change equation:

$$= \frac{(X2 - X1)}{|X1|} * 100$$

Optimization Method	Time to Conversion (days)	Percent Change
Standard Conditions	26	
Solid Support System	21	19.23% decrease[1]
Media Screening	14	33.33% decrease[2]
Inoculation with Fungal Mass	7	50.00% decrease[3]
**Overall Percent Change in Reaction Time Following Optimization**		**73.08% decrease[4]**

[1] =((21-26)/|26|)*100; [2] = ((14-21)/|21|)*100; [3] = ((7-14)/|14|)*100; [4] = ((7-26)/|26|)*100

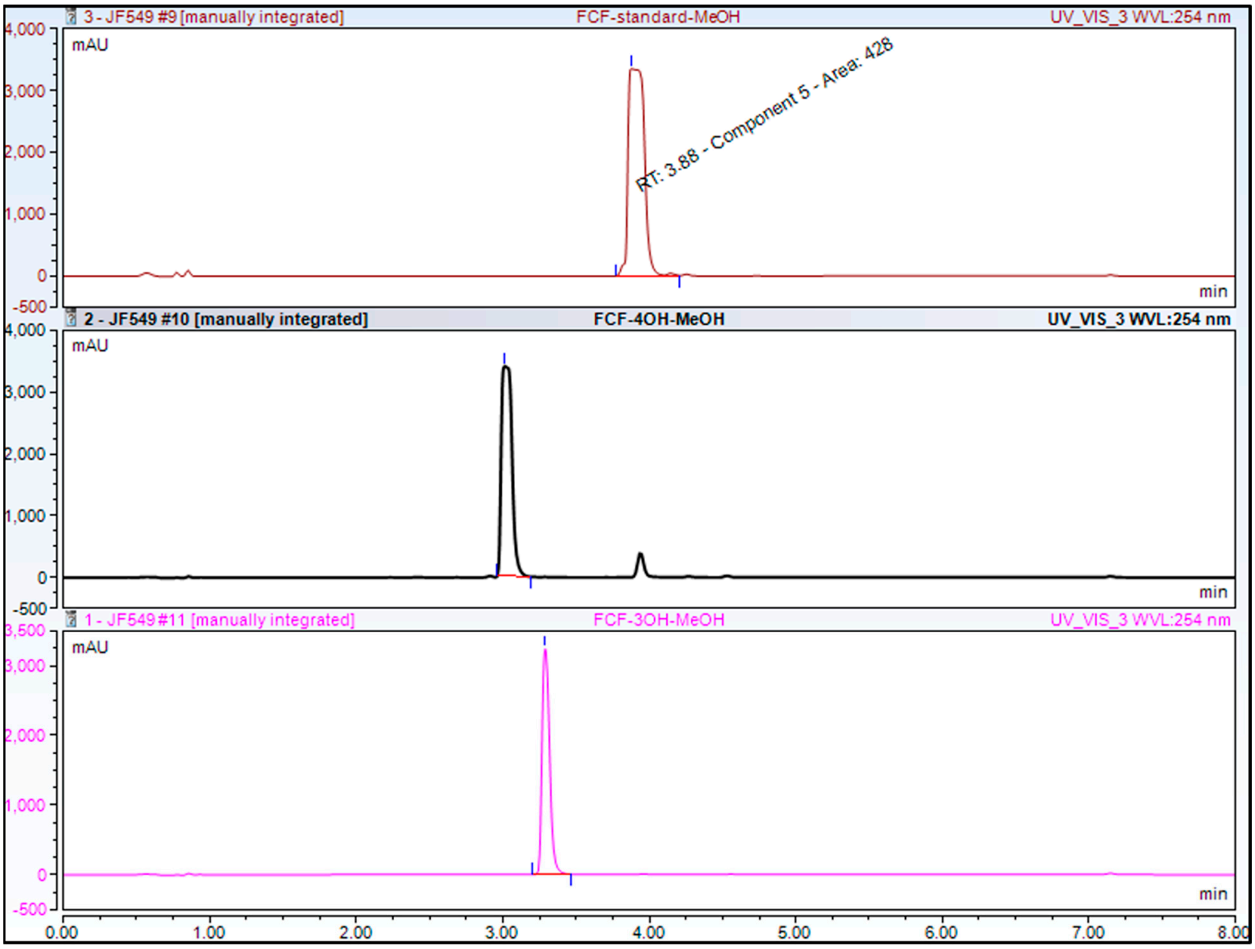

Figure S4. HPLC chromatogram of pure forchlorfenuron, 4-hydroxyphenyl-forchlorfenuron, and 3-hydroxyphenyl-forchlorfenuron overlay.

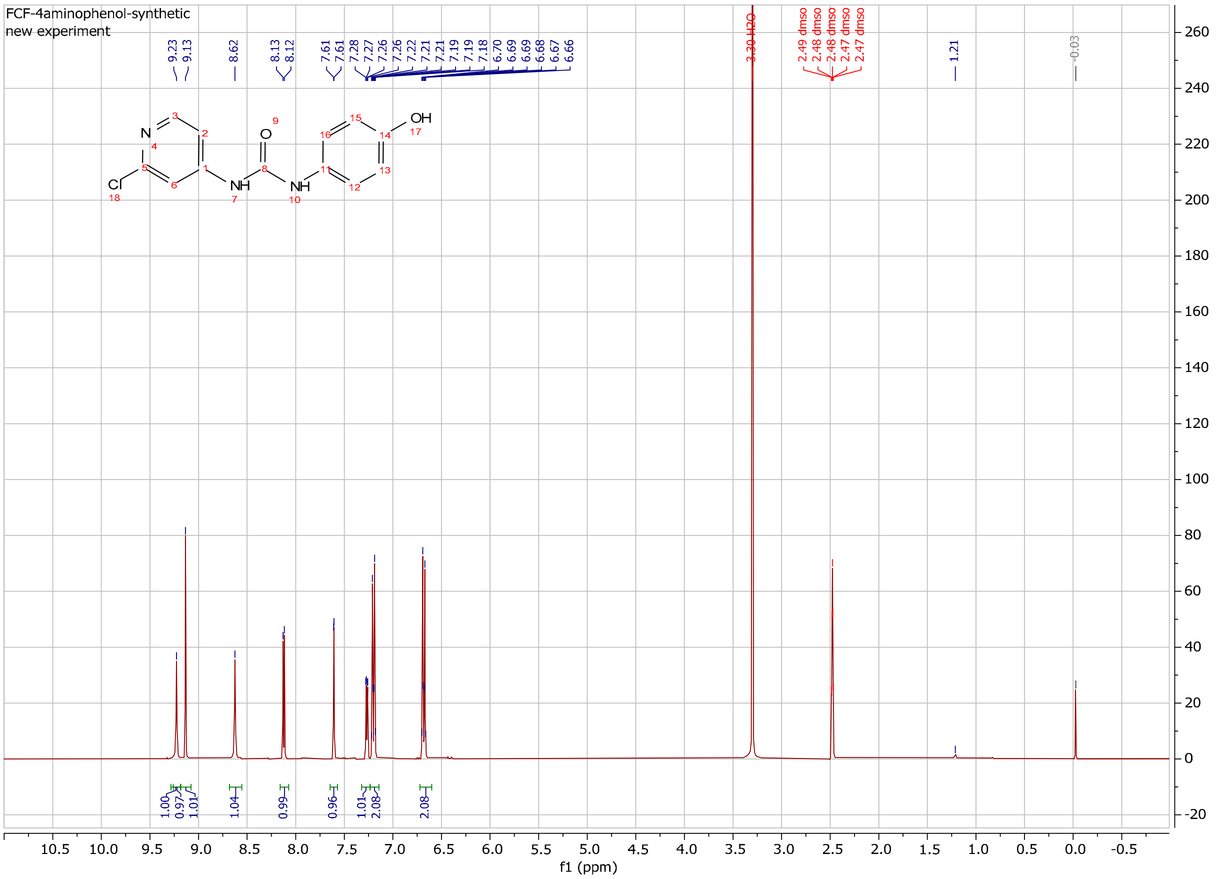

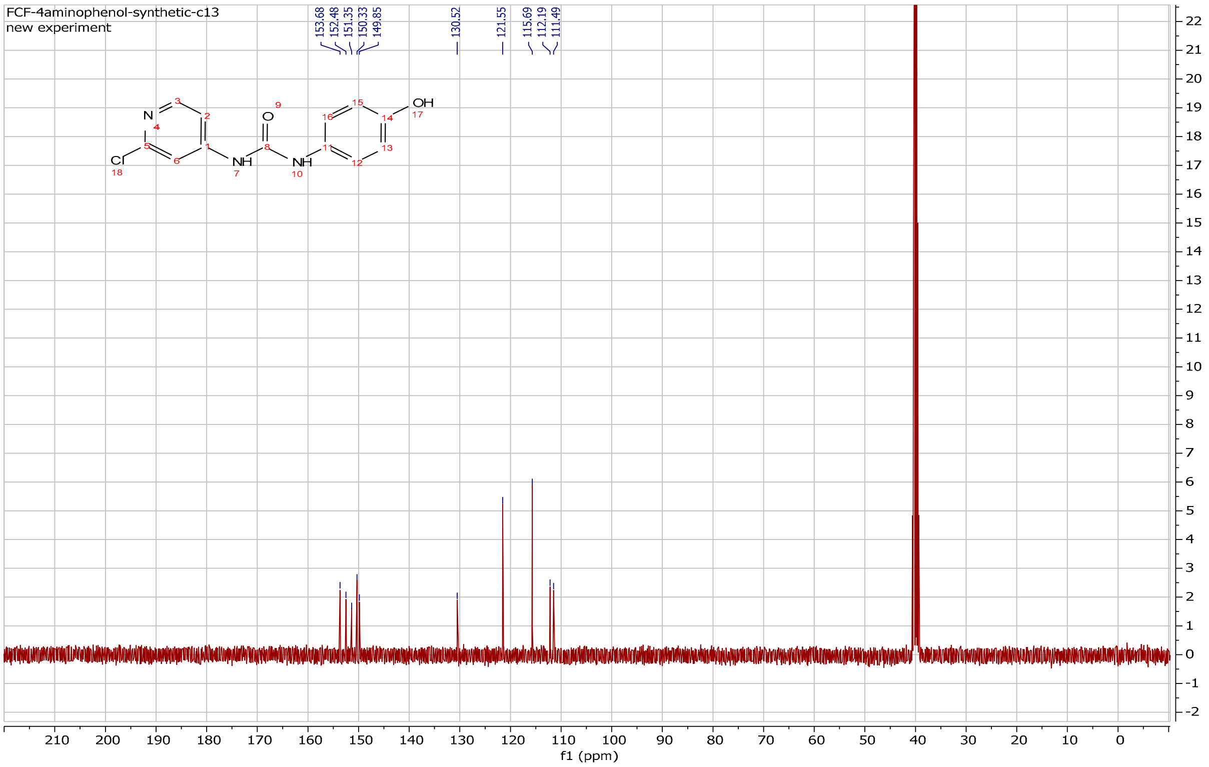

Figure S5. ^{1}H NMR and ^{13}C NMR data for the isolated metabolite (4-hydroxyphenyl-forchlorfenuron) found in the optimized biotransformation study.

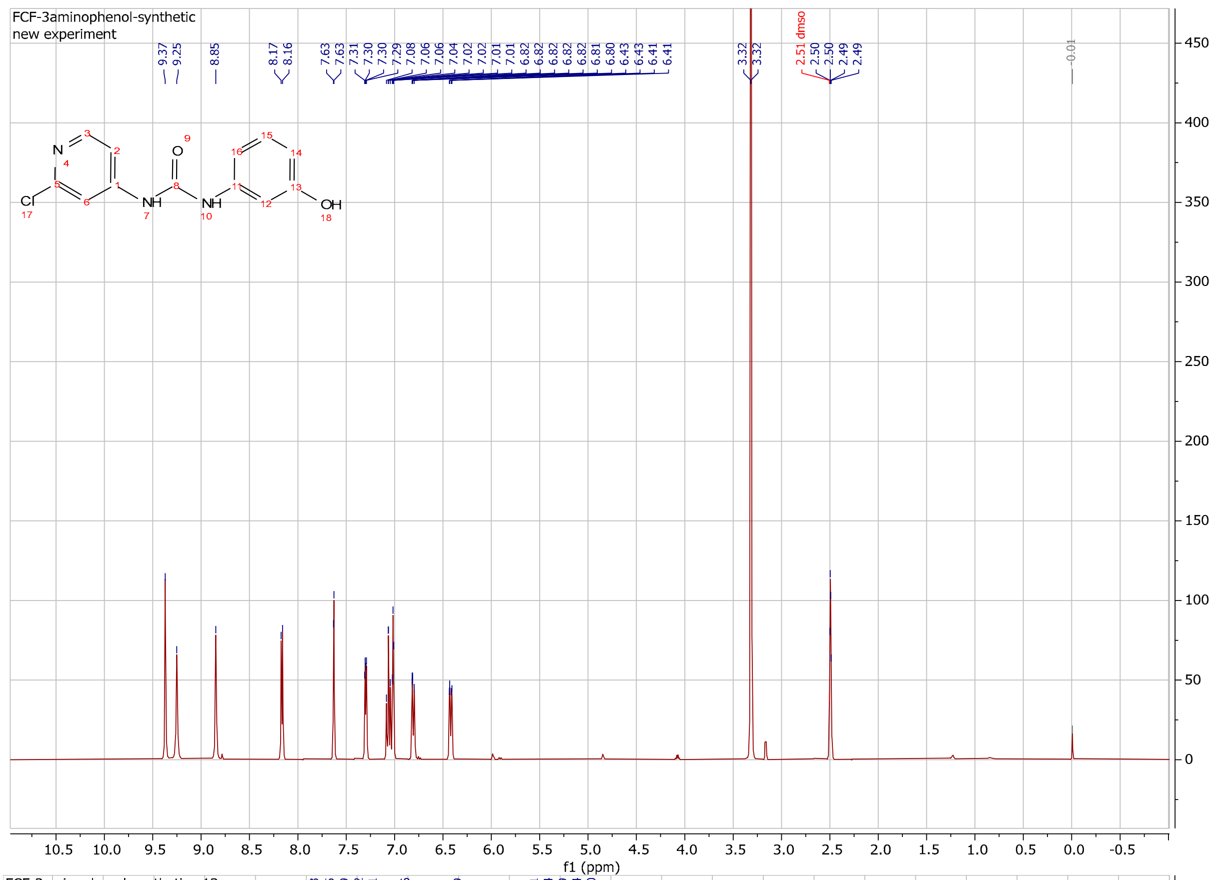

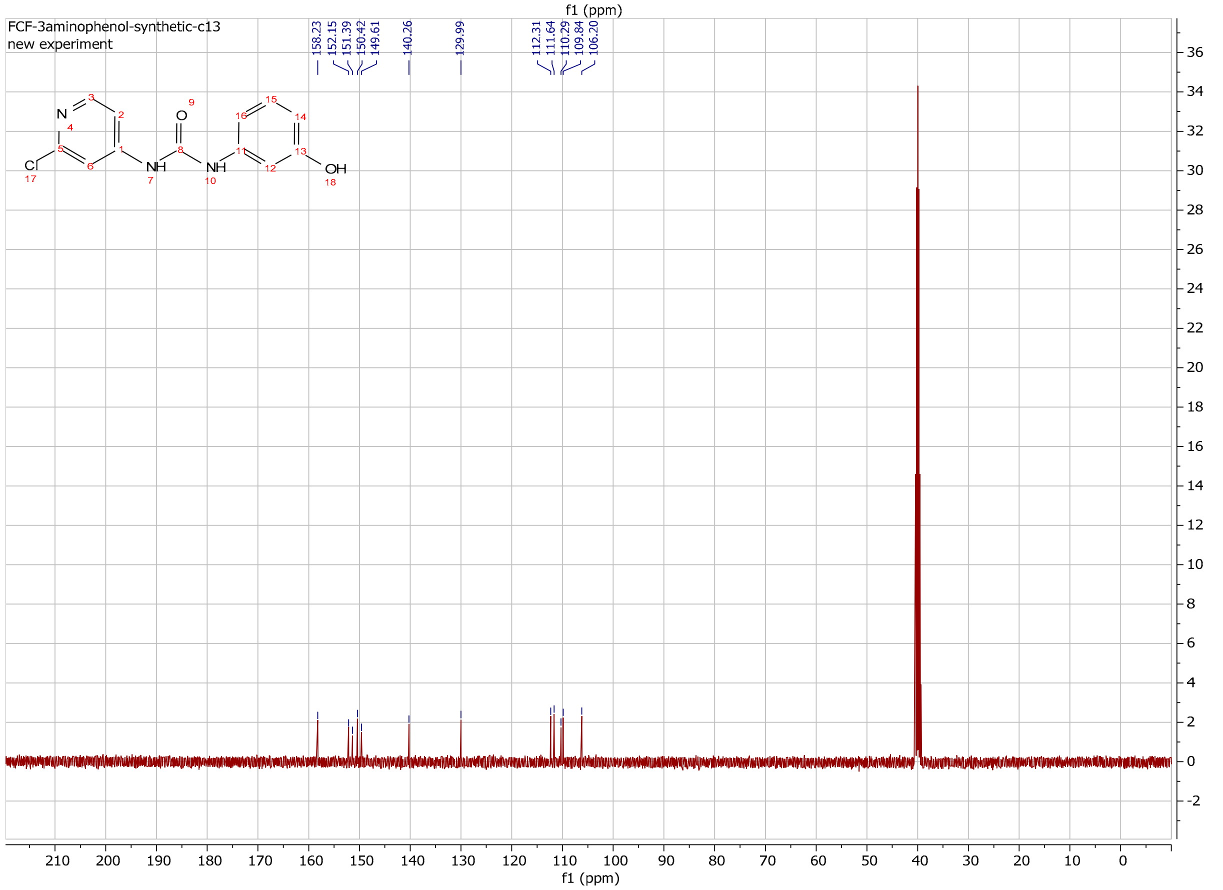

Figure S6. ^{1}H NMR and ^{13}C NMR data for the synthesized metabolites used as reference standards (4-hydroxyphenyl-forchlorfenuron and 3-hydroxyphenyl-forchlorfenuron).
